# The Effects of the Addition of Polyurethane–MgO Nanohybrids on the Mechanical Properties of Ordinary Portland Cement Paste

**DOI:** 10.3390/nano12223978

**Published:** 2022-11-11

**Authors:** Yu Fang, Weiqing Ning, Yuan Li, Fang Li, Reza Pournajaf, Bejan Hamawandi

**Affiliations:** 1 College of Urban Construction, Xi’an Siyuan University, Xi’an 710038, China; 2 Advanced Materials Research Center, Department of Materials Engineering, Najafabad Branch, Islamic Azad University, Najafabad 85141-43131, Iran; 3 Department of Applied Physics, KTH Royal Institute of Technology, SE-106 91 Stockholm, Sweden

**Keywords:** ordinary Portland cement, polyurethane, MgO nanohybrids, mechanical properties

## Abstract

One of the most important methods of controlling the properties of concrete and cement-based materials is to control the rate and kinetics of cement hydration. In the present study, novel flexible polyurethane-decorated MgO nanohybrids were synthesized using a simple chemical method, added to cement paste in different amounts, and utilized as an effective mechanical performance-enhancing factor for cement paste. It was observed that by adding 3 wt% synthesized PU-MgO nanohybrids to cement paste, its mechanical properties were improved and its compressive strength and flexural strength were increased by up to 13% and 15%, respectively, compared to the plain cement, after 45 days. The effect mechanism of adding PU–MgO nanoparticles on the properties of the cement paste was investigated. The addition of PU–MgO nanohybrids increased the pozzolanic reactions and formed more C-S-H phases.

## 1. Introduction

During the last 10 years, significant studies have been conducted for manipulating the structures of cement hydration products and the mechanical properties at the nano-scale using various nanomaterials, e.g., nano-SiO_2_ [1,2,3,4,5], nano-TiO_2_ [6], and nanotube- and graphene-based materials [7,8,9,10]. The improvement in mechanical properties is attributed to density and nucleation-site enhancement after the addition of nanomaterials [11,12,13]. As a result of the pozzolanic reaction of nano-MgO, a higher degree of polymerization interactions takes place in the calcium–silicates–hydrates phases, resulting in a condensed microstructure [14]. The use of different nanomaterials in cement materials has been expanded, but there are still many problems that require detailed research to overcome these obstacles. Nanomaterials are usually added to cement or concrete products directly, without additional processes. The properties obtained from the addition of conventional nanomaterials cannot be generalized for all cement products because conventional nanomaterials can agglomerate quickly due to their high specific surface area. This can cause accumulation and non-uniformity in the distribution of nanomaterials in the cement matrix. By agglomerating nanomaterials, cement-derived materials can be distributed unevenly, which is effective in improving the repeatability of their mechanical properties [15,16,17,18].

For low-carbon industries and sustainable development, inorganic-organic nanocomposites can enhance the mechanical features of cement materials. Nanomaterials can be modified with polymers to reduce agglomeration and increase pozzolanic interaction. In hydrated cement pastes, the C-S-H phase determines many characteristics, including the mechanical properties [19,20,21]. It is possible, however, that polymers with relatively neutral functional groups such as polyethylene glycol might not be able to form efficient surface interactions with hydrated phases [22]. Thus, organic-inorganic hybrids can be enhanced by selecting polymers with active groups. Flexible polyurethane (PU) possesses great mechanical properties compared to other polymers—such as styrene-butadiene rubber (SBR), ethylene vinyl acetate (EVA) copolymer, and styrene–acrylate latexes (SSA)—as well as high strength and toughness [23,24,25]. In a study conducted by Tang [26], it was found that the addition of polyurethane increased the compressive strength and durability of mortar in its early stages. Different functional groups in the molecular chain of polyurethane give it a flexible structure, which enables the molecular design of inorganic-organic nanocomposites with desired properties. The properties of polyurethane make it an excellent adhesive and coating material for corrosive environments, as well as being flexible at low temperatures, abrasion-resistant, and having variable hardness. As mentioned, polyurethane is used as an anti-corrosion coating for metal surfaces and protective coatings due to its properties [27,28,29]. Its low cost, film-forming properties, and high stability against acids and bases make this material suitable for these applications [27]. These characteristics are derived from the structure of polyurethane; the presence of an aromatic ring causes heat and chemical resistance, its hydroxyl sphere causes high adhesive properties, while its long hydrocarbon (aliphatic) chain gives this compound the ability to be flexible [30,31]. Several studies have demonstrated the use of nanosized inorganic fillers—such as nano-silica [32,33,34], micro-clays [35,36,37], nanosized TiO_2_ particles [38,39,40], and carbon nanotubes [41,42,43] to further improve the properties of polymers. It is not yet known whether nano-PU-MgO can affect cement.

In any case, one of the main goals to increase the mechanical strength of cement is to reduce the volume of its consumption for environmental and economic reasons. Moreover, nanosized particles can reduce the porosity in cement paste with their filling properties and improve the effectiveness of polymer filler reinforcement by choosing a suitable combination. Based on the previous literature, adding nanoparticles as a substitute for the concrete mix can help to increase the mechanical strength of cement. In addition, the use of nanoparticles instead of fine aggregates may aid in this objective. Even though MgO nanoparticles are introduced as an ideal nano-additive for cement, the combination of MgO nanoparticles with cement has received little attention in previous papers. In addition, there is no explanation of the combined effects of MgO nanoparticles and polymer compounds in improving the mechanical properties of cement in the existing literature. To fill this research gap, we investigated the effects of adding combinatorial nanoparticles on the mechanical properties of cement. In this study, nanosized PU-MgO nanohybrids were synthesized and then added to the cement paste in different amounts. The purpose of this research was to investigate the effects of adding PU-MgO nanohybrids on the properties of cement paste—especially its mechanical strength. The performance of the PU-MgO nanohybrids was evaluated by determining the cement paste’s microstructures, mechanical properties, mineral-phase composition, and calorimetric cement hydration heat. Moreover, this study discusses the probable mechanisms of the PU-MgO nanohybrids’ function in cement pastes.

## 2. Materials and Methods

### 2.1. Materials

MgO (99.9%) nanopowder was purchased from US-nano Co. Toluene (99.5%), ethanol (99.8%), γ-aminopropyltriethoxysilane (KH550, 99%), diphenylmethane diisocyanate (MDI), and PEG ether 2000 were purchased from Sigma-Aldrich, USA.

### 2.2. Synthesis of PU-MgO Nanohybrids

**Figure 1:**
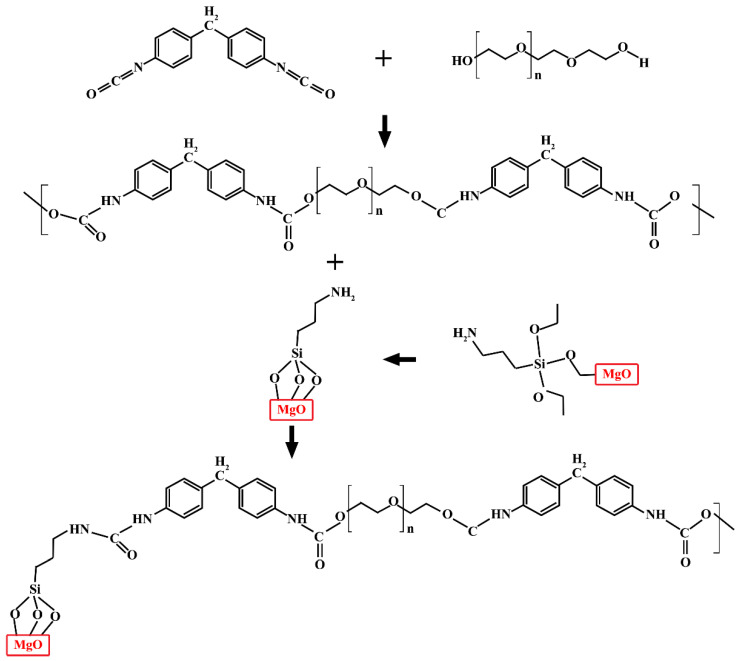
The schematic of the polymerization reaction between methylene diphenyl isocyanate (MDI) and the relatively long and flexible PEG. The MgO nanoparticles were functionalized by using γ-aminopropyltriethoxysilane (KH550) for anchoring amine groups on the surface to improve the interface between the MgO and PU.

The simplest and most widely used method for the synthesis of polyurethane is the use of polyol and isocyanate. After mixing polyol and isocyanate, the mixture is poured into the mold, and by gradually heating the mixture an elastomeric structure is created. To obtain an elastomer, raw materials can be used whose reaction leads to the production of a linear structure; This method is called one-step synthesis [44,45]. In this method, after mixing the raw materials, the reaction starts by increasing the temperature. Figure 1 shows the schematic of the synthesis procedure and the chemical structure of the PU-MgO nanohybrids, which were drawn using ChemDraw software. The PU-MgO nanohybrids were synthesized as follows: under anhydrous conditions, 40 g of MDI and 80 g of PEG2000 were reacted at 70 °C for 2 h.

After dissolving 20 g of nano-MgO (40 nm) in 500 g of ethanol, the mixture was sonicated at 25 °C for 20 min. At 50 °C, a solution of γ-aminopropyltriethoxysilane (KH550) was added dropwise to the aforementioned solution. Then, after dripping was performed, the produced mixture was incubated at 80 °C for 3 h. To obtain silane-coupled MgO, the reaction product was separated by centrifugation and dried. Then, 20 g of PU was added to 20 g of silane-functionalized MgO and 100 g of toluene in a three-necked flask and allowed to react at 110 °C for 5 h. After the reaction, centrifugation and a drying step were performed to obtain the product from the reaction mixture.

### 2.3. Production of Cement Pastes

Table 1 and Table 2 summarize the chemical composition and phases of the cement phase, respectively. The dosages of the PU-MgO nanohybrids in the cement pastes were 0, 1, 3, and 5 wt% (water/cement ratio = 0.38). The samples were prepared as follows: First, 3000 g of cement containing PU-MgO nanohybrids was poured into the mixer and thoroughly mixed at low speed for 4 min to ensure the fair distribution of the nanohybrids in the cement matrix. After adding 1140 g of water, the mixture was stirred for an extra 4 min. The PU medium is comparatively sticky as well as susceptible to water in the air. Therefore, adding intermediate PU directly to the cement paste to be used as a reference sample was challenging.

### 2.4. Mechanical Tests

An automatic cement paste strength testing machine was used to test the mechanical properties of the cement pastes. A mold measuring 40 × 40 × 160 mm^3^ was used to harden the cement paste samples. The flexural and compressive strengths of the samples were examined after the samples were treated in water for 3, 7, 28, and 45 days. The three-point bending technique, sans extra cutting, was used to directly test the cement paste specimens. The test complied with the GB/T 17671–1999 standard for determining the strength of cement (ISO method). Three samples were used to calculate the flexural strength, and six were used to calculate the compressive strength.

### 2.5. Characterizations

To determine the cement’s hydration heat curve, a TA Instruments TAM-AIR microcalorimeter (USA) was used. The 24-h equilibration and stabilization of the microcalorimeter were performed at 20 °C in advance. In the cement paste, the water-cement ratio was 0.38, and the polymer content was 0, 1, 3, and 5 wt%. A 20 mL plastic bottle containing 13.8 g of cement paste was sealed, and the exothermic heat was recorded for testing. A total interval of 150 h was required for the test.

Fourier-transform infrared spectroscopy (FTIR) was performed between 4000 and 400 cm^−1^ using a PerkinElmer FTIR device (USA). A transmission electron microscope (TEM; Philips model CM120, the Netherlands) was also used in this study. Phase characterization was carried out using X-ray diffraction (XRD) (Philips model PW3040, the Netherlands) at 40 kV and 25 mA, using Cu-Kα radiation (1.54184 Å). The chemical composition of the cement paste was measured using an X-ray fluorescence (XRF) analyzer (Shimadzu, model EDX-700, Japan).

## 3. Results and Discussion

### 3.1. Characterization of PU-MgO

**Figure 2:**
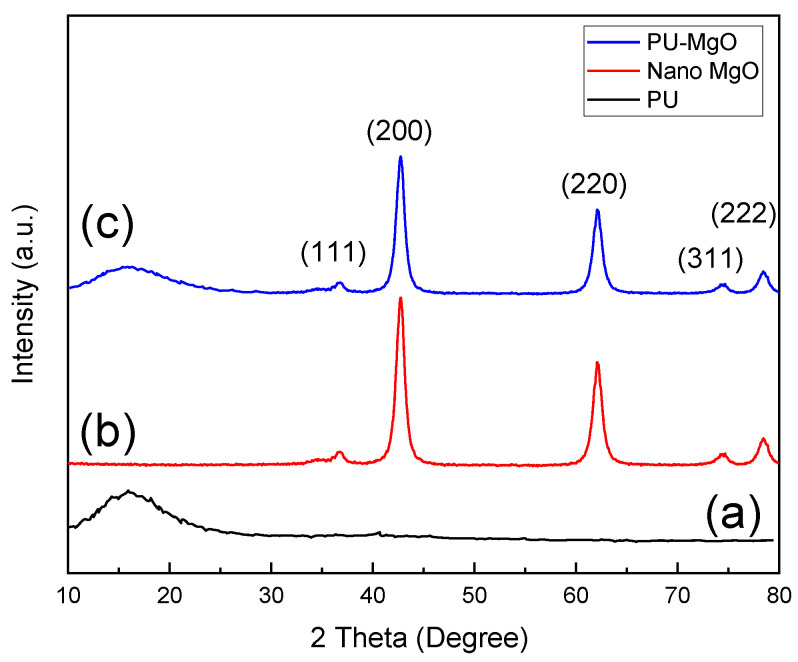
XRD patterns of (**a**) PU, (**b**) MgO, and (**c**) PU-MgO nanohybrids.

Figure 2a shows the XRD pattern of the PU, where a broad peak at ~20° suggests the amorphous behavior of the polyurethane [46]. Figure 2b presents the XRD pattern of the MgO powder. The results indicate that the whole peaks are linked to the standard cubic MgO reflections (JCPDS 45-0946). The diffraction peaks at 36.65°, 42.53°, 61.85°, 74.0°, and 78.4° correspond to the (111), (200), (220), (311), and (222) MgO crystal planes, respectively [47]. Figure 2c presents the XRD pattern of the synthesized PU-MgO nanohybrids, where every peak associated with the structure of MgO and the peak of ~20° related to the PU structure can be seen. It can be seen that the intensity of the peaks of MgO and PU in the nanohybrids was reduced due to the formation of the hybrid structure and the coverage of the peaks of the two compounds.

**Figure 3:**
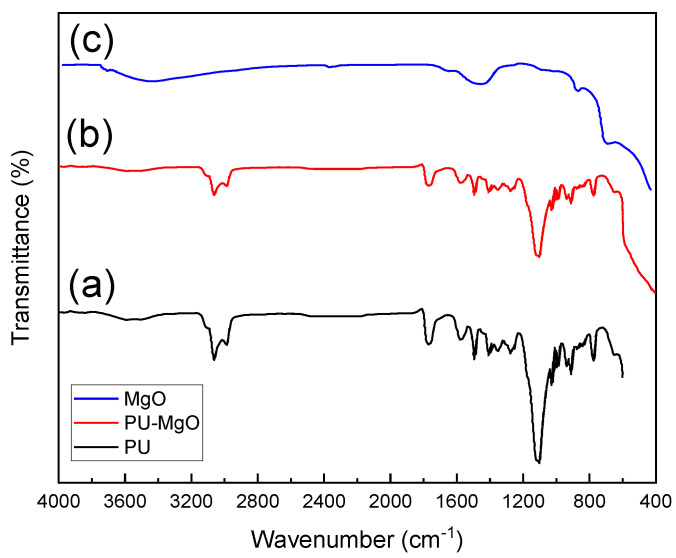
FTIR spectra of (**a**) PU, (**b**) PU-MgO, and (**c**) MgO nanohybrids.

The FTIR spectra of PU, MgO, and PU-MgO nanohybrids are shown in Figure 3. On the PU spectrum, the –CH_2_– stretching bands were found between 2926 and 2855 cm^−1^ [48], while the urethane carbonyl band was seen at 1706 cm^−1^ [1,46,49]. The C–N stretching bands, combined with those of the in-plane bending of N–H, were mostly seen at 1524 cm^−1^ and 1449 cm^−1^, showing the reaction between the hydroxyl group and the isocyanate [46]. The characteristic C–O–C band was seen at 1074 cm^−1^ [46,50]. In the MgO FTIR spectrum, O–H bond vibrations of the hydroxy group were observed in the broadband near 3696 cm^−1^. MgO nanoparticles were indicated by the peak at 421 cm^−1^ [51,52]. At 1386 cm^−1^, vibrations associated with C–H bending were detected [52]. The peaks seen at 1741 cm^−1^ and 1644 cm^−1^ indicated the presence of strong C=O and C=C stretching, while the peak at 3261 cm^−1^ showed the existence of weak, broad O-H bond stretching [46]. In the spectrum of the PU-MgO nanohybrids, peaks related to PU and MgO were observed.

**Figure 4:**
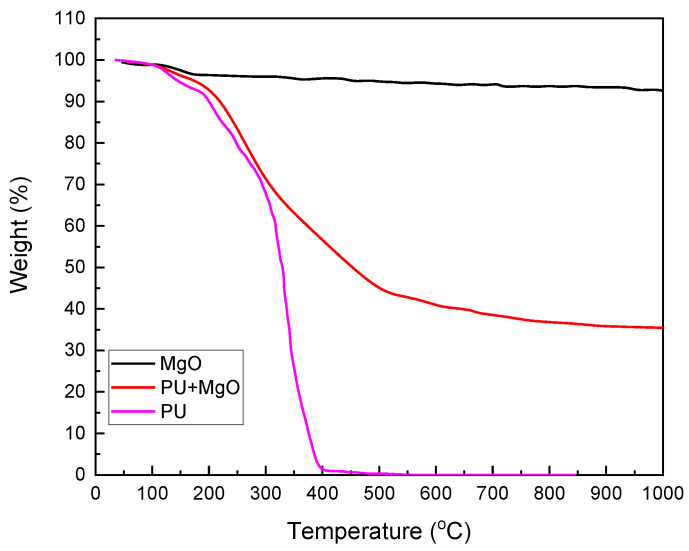
TGA curves of PU, MgO, and PU-MgO nanohybrids.

Figure 4 presents the TGA curves of the PU, MgO, and PU-MgO nanohybrids. In the thermogram related to nano-MgO, a total weight loss of about 5% was observed at temperatures lower than 200 °C. This weight loss was related to moisture and carbon dioxide absorbed from the environment and has also been reported in other studies on MgO [53,54]. As can be seen, pure polyurethane is completely burned above 410 °C. From the beginning of heating, up to about 100 °C, moisture evaporates. After that, the degradation of polyurethane bonds starts up to 200 °C, and this probably also leads to the decomposition of isocyanate and alcohol, producing primary amines, olefins, and secondary amines. In the next step, ester bond dissociation occurs and, finally, hydrocarbon chains and siloxane components are decomposed [55]. The thermogram of the nanohybrids sample showed the degradation profile in the form of three stages, with the most significant decrease in weight occurring in the secondary phase.

At the beginning of the decomposition, evaporation of the trapped moisture and solvent was observed. First, urethane bonds were decomposed. During this stage of decomposition, isocyanates and alcohol decompose to produce primary amines, olefins, and secondary amines. The second stage of decomposition was due to the dissociation of ester bonds, while the decomposition of hydrocarbon chains and siloxane components was observed in the third stage [46,55]. Similar types of decomposition steps have been reported. It is also reported in the literature that adding nanoparticles to polyurethane increases its thermal stability and storage modulus profiles. As a result of hydrogen bonding with the polyurethane matrix and urea groups within the nanoparticles, solid network structures were formed. These acted as a thermal insulators and mass-transfer barriers. Decomposition produces volatile products, which increase the temperature [55,56].

**Figure 5:**
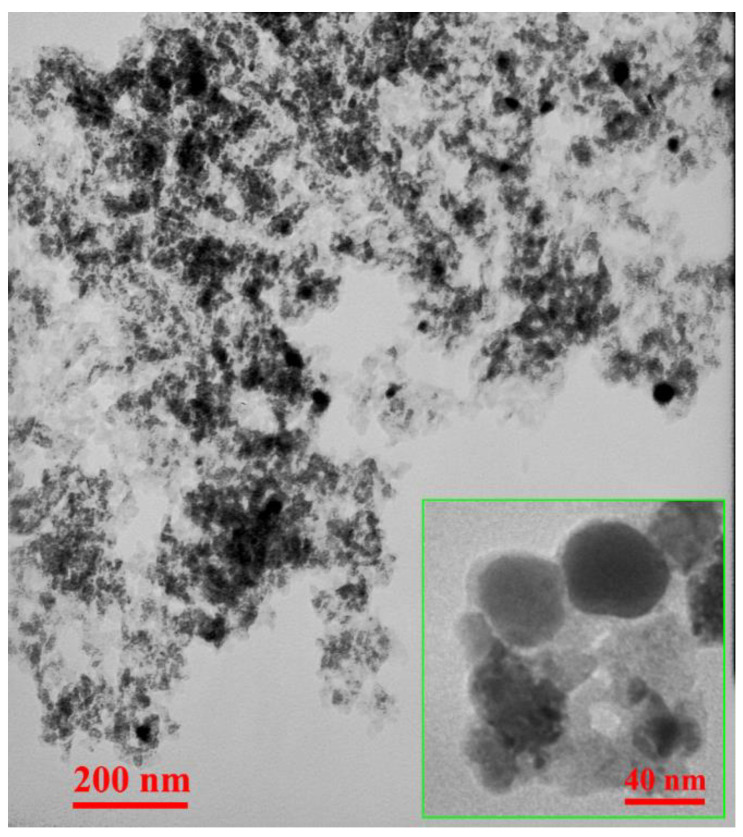
TEM micrographs of PU-MgO nanohybrids.

Figure 5 shows the TEM micrographs of the PU-MgO nanohybrids, indicating their morphology, shape, and distribution characteristics. Hydrophilic PU (PEG2000) was found in a number of small nanohybrid agglomerates. Polyurethane’s hydrophilicity and hydrophobicity may influence the distribution of nanohybrids in a cement matrix, thereby affecting the final properties of the cement paste.

### 3.2. Mechanical Properties

**Figure 6:**
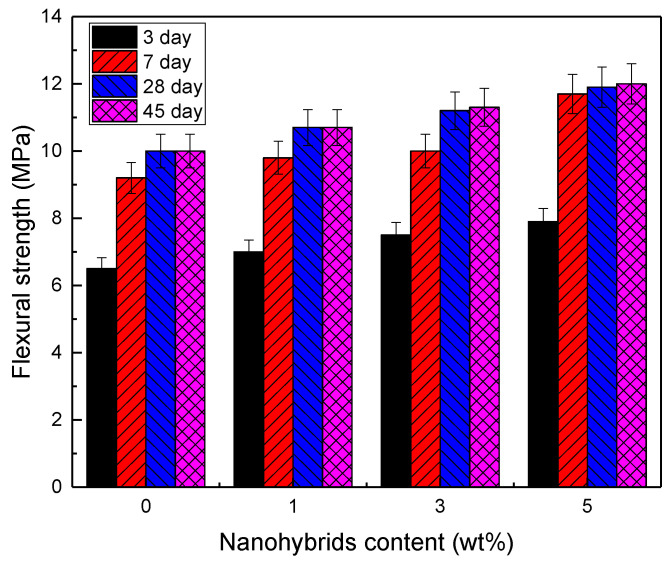
Flexural strength of cement pastes with different nanohybrid contents at 3, 7, 28, and 45 days.

Table 3 and Figure 6 show the flexural strength of the prepared cement paste with different nanohybrids and cured for 3, 7, 28, and 45 days. The flexural strength of plain cement at 7 and 28 days was significantly higher than at 3 days of curing. Despite this, the flexural strength of the cement paste did not change at 45 days. The flexural strength of the cement pastes was enhanced by adding the PU-MgO nanohybrids. Cement pastes mixed with 3 wt% nanohybrids had a flexural strength of 7.5, 10, 11.5, and 11.8 MPa after curing for 3, 7, 28, and 45 days, respectively, representing increases of 14%, 8%, 14%, and 15% compared to plain cement paste, respectively. Since the addition of nanoparticles fills microcracks and voids, it significantly improved the structure of the composite matrix. Nanoparticles act as an external nucleation site in the matrix, accelerating the formation of C-S-H gels, thereby increasing the amount of C-H crystal, which was the determining factor of flexural strength. The degree of increase in flexural strength caused by the presence of nanoparticles was related to the nucleation efficiency and the formation of phases caused by it. The results showed that the addition of more than 3 wt% nanoparticles did not significantly change the strength increase rate. As a result of increasing the nanoparticle content, the flexural strength rate was decreased due to their agglomeration in the microstructure. Similar results for other nanoparticles have been reported for obtaining the optimal flexural strength in the literature [57,58].

**Figure 7:**
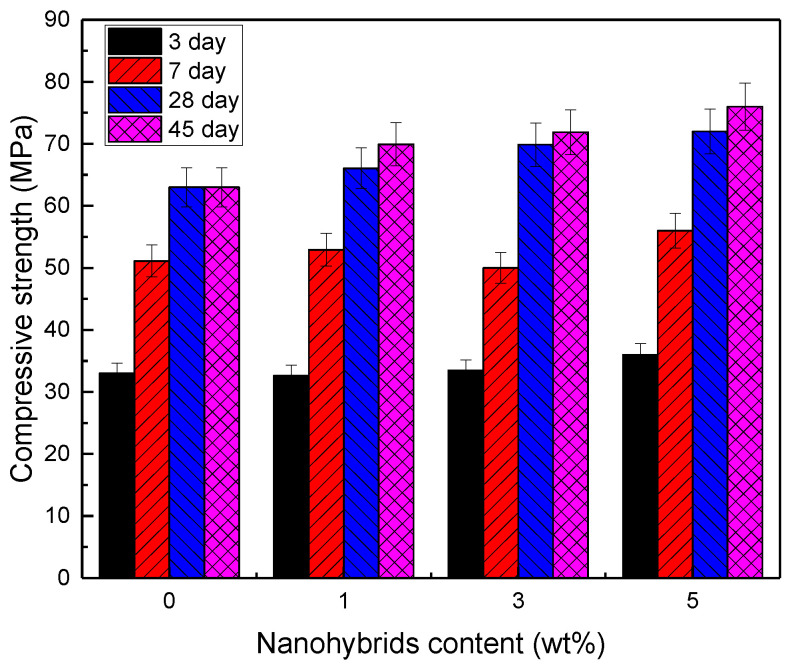
Compressive strength of cement pastes with different nanohybrid contents at 3, 7, 28, and 45 days.

The compressive strength of the cement pastes with different nanohybrid contents after 3, 7, 28, and 45 days of curing is shown in Figure 7 and Table 3. A noticeable increase in compressive strength was also observed in the cement pastes containing nanohybrids. After 3, 7, 28, and 45 days of curing, the cement pastes with 3 wt% nanohybrids had compressive strengths of 33, 50, 70, and 72 MPa, respectively, representing increases of 4.5%, 2%, 10%, and 13%, respectively. The improvement in compressive strength with the addition of nanoparticles can be attributed to the pozzolanic reaction of nano-MgO, reacting with C-H crystals to produce an additional C-S-H gel, which was the primary source of the compressive strength of the cement-based materials. Furthermore, the MgO nanoparticles can fill the nano-voids and pores in the C-S-H gel, thereby improving the density and micro/nano-packing [57]. MgO nanoparticles act as nucleation sites and create a strong bond with the C-S-H gel particles. As a result, they improve the stability of the hydration products [59].

**Figure 8:**
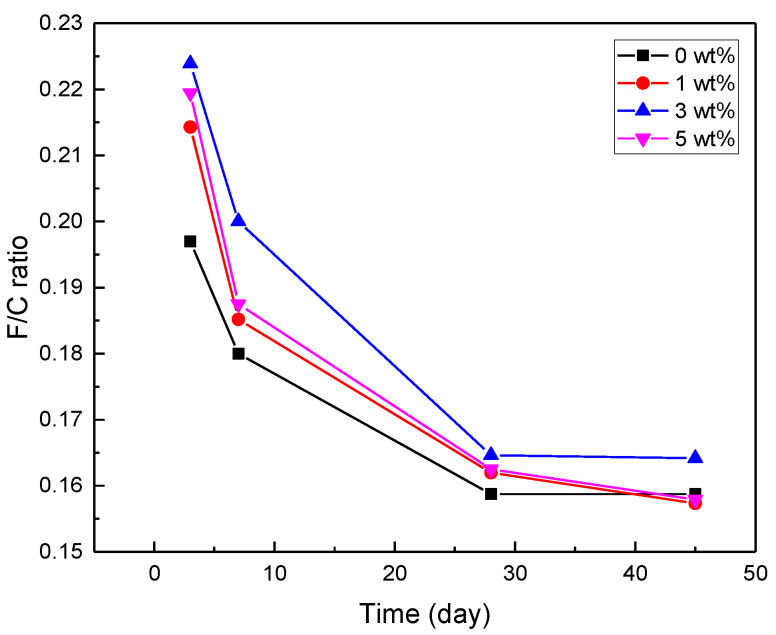
F/C ratio of cement pastes with different nanohybrid contents at 3, 7, 28, and 45 days.

According to Figure 8, cement pastes containing 3 wt% nanohybrids presented a higher flexural-to-compressive strength (F/C) ratio than the other samples at various ages, thus enhancing the flexural properties of the cement-based materials. The early compressive strength of the cement pastes enhanced after 7 days when MgO was added to them, which resulted in a lower F/C ratio than the plain cement paste after 7 days. When increasing the hydration time from 3 to 45 days, all of the cement pastes’ F/C ratios decreased significantly. The rapid increase in the cement pastes’ compressive strength from 3 days to 45 days accounts for this phenomenon. However, this period also saw a gradual increase in the cement pastes’ flexural strength [1]. Overall, it can be said that with an increase in the nanoparticle content up to 3 wt%, the value of F/C increased, indicating the positive effect of the nanoparticles on the flexural strength of the cement pastes; meanwhile, with a further increase in the nanoparticle content, the improvement in the compressive strength of the cement paste was more evident.

### 3.3. Phase Characterization

**Figure 9:**
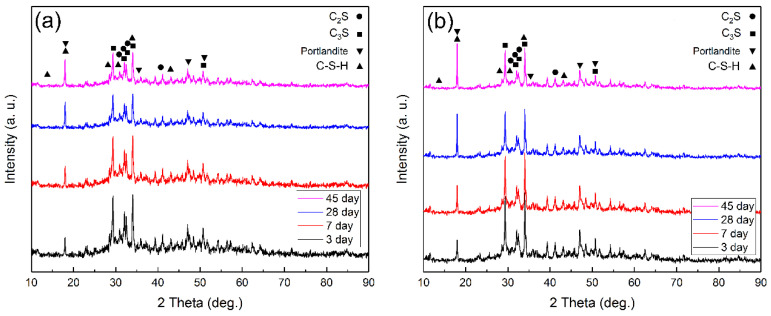
XRD patterns of cement pastes at 3, 7, 28, and 45 days with different nanohybrids: (**a**) 0 wt% and (**b**) 3 wt%.

Figure 9 shows the XRD patterns of the cement pastes with different nanohybrid contents after 3, 7, 28, and 45 days of curing. To compare and investigate the impact of the produced nanohybrids on the phase structure of the cement pastes, phase studies of cement paste samples without nanohybrids and with 3 wt% nanohybrids were carried out.

The 2θ peaks of 32.1°, 32.45°, 51.75°, 29.3°, and 34.2° are the most intense peaks related to the C_3_S with the standard card number (00-049-0442), corresponding to the (003), (22-2), (62-2), (22-1), and (221) crystal planes, respectively. The peaks at 32°, 32.6°, 32.1°, and 41° were also observed, corresponding to the crystal planes (10–3), (200), (12–1), and (031), respectively, of the C_2_S phase with the standard card number (00-049-1673). The phase resulting from the hydration of cement was the C-Si-H phase, and the peaks at the angles of 28°, 31°, 13.6°, 32.6°, 17.5°, and 41.8° were related to this phase with the standard card number (00-003-0510). The peaks related to the portlandite phase also appeared at angles of 34.6°, 18°, 47°, and 51°, which correspond to the (101), (001), (102), and (110) planes, respectively, with the standard card number (00-001-1079). In general, the formation of C-S-H phases starts slowly in the early stages. As the primary reaction begins, more C-S-H and Ca(OH)_2_ gels are formed, leading to a standard setting. As seen in the XRD pattern of the cement paste sample without nanohybrids, the peak related to the portlandite phase increased with age, but the C_2_S and C_3_S phases could still be seen in the sample, and the intensity of the peaks of these two phases decreased with age. With the addition of PU-MgO nanohybrids, the peaks related to the C_2_S and C_3_S phases decreased more than in the plain sample, and the portlandite phase and other hydrated phases increased significantly—especially in the later stages. This phenomenon shows an increase in the kinetics of pozzolanic reactions and the formation of hydrated phases [26,60,61]. The formation of these phases led to an increase in strength, which is entirely consistent with the results obtained from the strength tests.

### 3.4. Cement Hydration

**Figure 10:**
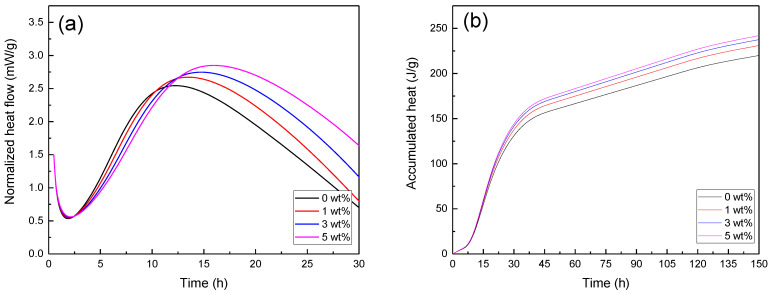
Hydration heat of cement paste with different nanohybrids vs. time: (**a**) instantaneous heat flow and (**b**) cumulative heat flow.

Figure 10 shows the curves of the hydration heat of cement pastes with various nanohybrid contents vs. time. By comparing the hydration heat of samples to various amounts of PU-MgO nanohybrids, it can be seen that the addition of PU-MgO nanohybrids did not cause an additional peak in the graph, and only its intensity was changed. In all samples, one peak was observed at the initial hydration times. The released heat increased with the increase in the amount of nanohybrids, which may have been due to the rapid formation of the brucite phase related to the nanohybrids [62]. The released heat was minimal because magnesium oxide was used in the hybrid condition, and most of its surface was covered by polyurethane. The central peak of the heat release for the plain sample reached the highest value after approximately 11 h. After that, the rate of hydration decreased steadily with the increase in the hydration time, along with a reduction in the released heat. It can be seen that with the addition of PU-MgO nanohybrids to the cement paste, the heat released was increased compared to the plain sample. This increase in heat release indicates a greater rate of reaction to form hydrated phases, but the addition of nanohybrids delayed some of the hydration reactions. In another investigation, this delay in hydration was related to the rapid crystallization and precipitation of Mg(OH)_2_ from the cement paste solution, reducing the concentration of OH^−^ ions. This delay increases when the Ca(OH)_2_ saturation ratio attains a peak; therefore, the onset of the secondary maximum in the cement’s heat evolution curve is postponed to the end of the induction time. Another reason is that brucite produced containing small crystallites can deposit on the surfaces of cement grains and form a thin layer that delays the hydration of most cement [62,63,64]. In the later stages, the heat released increases with the increase in the nanohybrid content, indicating the formation of more hydrated phases. This phenomenon led to the achievement of higher strength in cement paste in the later stages.

Essentially, the setting of cement is based on the hydration of calcium silicate phases in the cement’s composition. The higher the degree of hydration, the more heat will be released by the reaction, leading to higher strength. Therefore, the study of cement’s setting provides important information. As mentioned, the addition of PU-MgO nanohybrids increased the heat released, but this was associated with a time delay in the later stages, showing that the addition of these nanohybrids to cement can have positive effects on the final properties of cement, as seen in the compressive strength test. These nanohybrids increased the strength of the cement.

In another similar study conducted by Ma et al. [1] on silica–polyurethane nanohybrids, it was found that during the setting of the cement, calcium ions were gradually released during the hydration process. These were absorbed due to electrostatic effects on the nanohybrids or the surface of the C-S-H phases. The results obtained from structural studies and thermometry of hydration for PU-MgO nanohybrids also showed that adding these nanohybrids to cement will increase hydration, especially in the later stages. The mechanism of action of these nanohybrids may also be the same as that of silica–polyurethane nanohybrids, in that the activity of the PU-MgO nanohybrids can increase the absorption of calcium ions and increase the hydration reaction kinetics, leading to the formation of more pozzolanic phases and, consequently, increasing the strength of the cement paste. It has been reported in many sources that the addition of large amounts of ordinary MgO can reduce the strength of concrete or cement paste because it can reduce the formation of C-S-H phases [65]. Moradpour et al. [66] observed that adding small amounts of MgO nanoparticles can hydrate MgO in the calcium silicate hydration process in the later stages.

**Figure 11:**
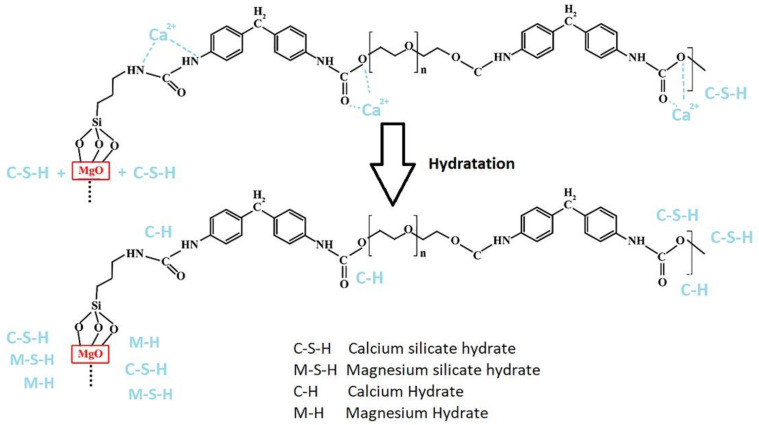
Mechanism of the effect of nanohybrids on cement hydration products.

Figure 11 shows the mechanism of the effect of the nanohybrids on cement hydration products. Moreover, Mg(OH)_2_ and magnesium silicate hydrate (M-S-H) are formed. The results confirmed that the crystallinity of C-S-H was enhanced in the specimen that contained nano-MgO compared to the plain sample. Furthermore, by altering the degree of crystallinity, the length of the silicate chain and the silicate density through the layer (crosslinking) for C-S-H in MgO-containing samples compared to those in the plain samples were increased more steeply. Therefore, adding a moderate amount of MgO to the cement-based composites led to improvement in mechanical properties because of the incorporation of moderate amounts of Mg^2+^ ions in the C-S-H nanostructure and the modification of the latter [62,67,68,69]. The results obtained for the PU-MgO nanohybrids showed a similar mechanism. Many polymers, including polyurethane, can absorb dissolved metal ions due to the presence of carboxyl and amino groups in their structure. In the synthesized nanohybrids, dissolved calcium ions can be absorbed by oxygen or nitrogen atoms of the polyurethane chain and then turned into calcium hydrate and participate in pozzolanic reactions. Various studies have reported the interaction between nitrogen and oxygen in the polymer chain as a factor in the absorption of soluble metal ions [70,71,72].

The presence of PU-MgO nanohybrids in the cement matrix can improve hydration in several ways. One is that the polyurethane polymer chain groups can cause the absorption of ions in the cement environment—especially the absorption of calcium ions on the nitrogen and oxygen atoms in the polyurethane chains. Then, these adsorbed ions can be hydrated or, by adsorbing on the surface of cement particles, they can cause the formation of primary nuclei and accelerate the processes of nucleation and hydration [70,71,72]. On the other hand, MgO nanoparticles themselves can also create M-S-H gels, improving the physical and mechanical properties of cement materials and their composites.

The surface of cement particles adsorbs H_2_O molecules and forms hydrated phases. The purpose of adding nanoparticles to cement is to stimulate nucleation processes during the initial hydration of the cement. The earlier these nuclei are formed, the earlier they can turn into larger crystals of hydration phases and, thus, accelerate the cement’s hydration [73]. Nanoparticles create a very large surface area due to their small size. At the same time, these surfaces are highly reactive and may react with pore solution components or may act as nucleation sites [74]. They provide large reactive surfaces that may act as nucleation sites, thereby stimulating the nucleation of the hydration phase on their surface. Nanoparticles also have great potential to react with cement paste components to form additional nuclei in a C-S-H pozzolanic reaction. Since the accelerating effects of these particles are induced by surface reactions, the surface area and particle size are the main factors in the particles’ efficacy in controlling the cement hydration kinetics.

One of the obstacles in this approach is the agglomeration of nanoparticles and their non-uniform distribution; to solve this problem, nanomaterials have been used in hybrid ways. In addition, some of the hybrid nanomaterials that have been used in recent studies include the hybridization of silica nanoparticles with polyurethane. It has been shown that the polyurethane chain groups can act as nucleation sites and create pozzolanic phases by absorbing dissolved ions [1]. In the work of Tang et al. [26], the positive effect of the addition of polyurethane on the cement properties was reported. On the other hand, MgO itself can create M-S-H gels, which can improve the physical and mechanical properties of cement materials and their composites.

## 4. Conclusions

In this study, nanohybrids of MgO and PU were synthesized. Then, different amounts of the PU-MgO nanohybrids were used as additives to ordinary Portland cement. The results showed that the sample containing 3 wt% PU-MgO nanohybrids had optimal properties. The results are as follows:

The flexural strength of cement pastes with the addition of 3 wt% PU-MgO nanohybrids increased by up to 15.0% after 45 days of curing compared to plain cement pastes. Additionally, after 45 days of curing, the compressive strength of the cement pastes increased by up to 13% with the addition of 3 wt% PU-MgO nanohybrids. Furthermore, cement pastes with PU-MgO nanohybrids have a higher prominence in hydration peaks compared to plain cement paste, leading to the formation of high-strength cement-based materials. A significant decrease in the quantity of C-H was caused by the pozzolanic activity of the PU-MgO nanohybrids, as well as the formation of C-S-H, which led to an increase in the cement paste’s mechanical strength.

The results showed that the addition of PU-MgO nanohybrids increased calcium ion absorption and improved hydration in the later stages. The increase in released heat indicated the increase in hydration and hydrated phases with a time delay, which was due to the rapid crystallization and precipitation of the brucite phase from the cement paste solution. In any case, it is recommended that the resistance of this cement paste should be checked after a few years.

## Figures and Tables

**Table 1:** Chemical composition of the cement used in this study.

Component	CaO	SiO_2_	Al_2_O_3_	MgO	SO_3_	Na_2_O	K_2_O	Fe_2_O_3_	L.O.I *
wt%	65	23.2	4.9	1.4	1.6	0.3	0.35	0.35	2.6

* Loss on ignition at 1000 °C.

**Table 2:** Chemical phases present in the cement.

Name	Abbreviation	Chemical Formula
Alite	C_3_S	Ca_3_SiO_5_
Belite	C_2_S	Ca_2_SiO_4_
Aluminate	C_3_A	Ca_3_Al_2_O_6_
Portlandite	P	Ca(OH)_2_
Ettringite	E	[Ca_6_[Al(OH)_6_]_2_·24H_2_O] [(SO_4_)_3_·1.5H_2_O]
Calcite	CaO	CaO
Calcium–Silicates–Hydrates	C-S-H	(CaO) × (SiO)-(2H_2_O)y

**Table 3:** Flexural strength, compressive strength, and F/C ratio of the samples with different nanohybrids.

Curing Time	Strength (MPa)	Nanohybrids (wt%)			
		0	1	3	5
3 Days	Flexural	6.5	7	7.5	7.9
	Compressive	33	32.6	33.5	36
	F/C Ratio	0.197	0.215	0.224	0.219
7 Days	Flexural	9.2	9.8	10	10.5
	Compressive	51	53	50	56
	F/C Ratio	0.180	0.185	0.200	0.187
28 Days	Flexural	10	10.7	11.5	11.7
	Compressive	63	66	69.8	72
	F/C Ratio	0.159	0.162	0.165	0.162
45 Days	Flexural	10	11	11.8	12
	Compressive	63	70	71.8	76
	F/C Ratio	0.159	0.157	0.164	0.158

## Data Availability

Not applicable.
